# Circadian glucocorticoids throughout development

**DOI:** 10.3389/fnins.2023.1165230

**Published:** 2023-04-25

**Authors:** Marianne Lehmann, Katharina Haury, Henrik Oster, Mariana Astiz

**Affiliations:** ^1^Institute of Neurobiology, University of Lübeck, Lübeck, Germany; ^2^Achucarro Basque Center for Neuroscience, Science Park of the UPV/EHU, Leioa, Spain; ^3^IKERBASQUE, Basque Foundation for Science, Bilbao, Spain

**Keywords:** circadian system, hypothalamic-pituitary-adrenal axis, development, perinatal programming, glucocorticoids, glucocorticoid receptor, molecular clock

## Abstract

Glucocorticoids (GCs) are essential drivers of mammalian tissue growth and maturation during one of the most critical developmental windows, the perinatal period. The developing circadian clock is shaped by maternal GCs. GC deficits, excess, or exposure at the wrong time of day leads to persisting effects later in life. During adulthood, GCs are one of the main hormonal outputs of the circadian system, peaking at the beginning of the active phase (i.e., the morning in humans and the evening in nocturnal rodents) and contributing to the coordination of complex functions such as energy metabolism and behavior, across the day. Our article discusses the current knowledge on the development of the circadian system with a focus on the role of GC rhythm. We explore the bidirectional interaction between GCs and clocks at the molecular and systemic levels, discuss the evidence of GC influence on the master clock in the suprachiasmatic nuclei (SCN) of the hypothalamus during development and in the adult system.

## 1. Circadian clock development and organization at systemic, circuit, and molecular level in the adult

Circadian rhythms are controlled by a clock system that conveys ~24 h rhythmicity to almost every physiological function in a living organism. When (and how) does the clock system really start ticking, and more specifically, which cells and mechanisms are involved, are long-standing questions in the field of chronophysiology. In adult mammals, circadian timekeeping is organized hierarchically with a central clock synchronizing subordinated clocks in all tissues to ensure that complex functions such as activity (Moore and Eichler, 1972), sleep (Collins et al., 2020), food intake (Nagai et al., 1978; Koch et al., 2020), and stress responses (Oster et al., 2006; Son et al., 2008) are efficiently adapted to a rhythmic environment.

The master pacemaker of the circadian system is located in the hypothalamic suprachiasmatic nucleus (SCN) (Lehman et al., 1987; Romero et al., 1993; Silver et al., 1996). Environmental factors that set the time (or entrain) internal clocks are called *zeitgebers* (from the German term for “time givers”). The main *zeitgeber* for the SCN is ambient light which reaches the retina and, in turn, the SCN through the retino-hypothalamic tract (RHT) (Ibuka et al., 1977; Hannibal et al., 2000).

The SCN has been traditionally described as a nucleus of around 20,000 neurons localized on each side of the third ventricle. It is topologically divided into “ventral core” and “dorsal shell” (Hastings et al., 2018). Neurons in the core receive direct photic input from the retina and transmit this signal to the surrounding shell. The neurons in the SCN shell, that in absence of light show autonomous activity with an endogenous period of ~24 h, are then entrained and translate their synchrony by efferent connections to subordinated clocks in multiple brain regions including, importantly, clocks within the medial hypothalamus, where key cell groups organize hormone release and set the tone of the autonomic nervous system (Dibner et al., 2010; Kalsbeek et al., 2011). Interestingly, this neuron-centered view changed recently when astrocyte clocks were found to be partially necessary and completely sufficient to restore neuronal circadian function in the SCN (Brancaccio et al., 2019). Rather than being “mere support cells” for the SCN neuronal circuit, they play an essential role as time-keepers of the adult master clock (Barca-Mayo et al., 2017; Brancaccio et al., 2017, 2019; Tso et al., 2017).

Glucocorticoids (GC) are examples of rhythmic hormonal outputs tightly regulated by the SCN and well-known mediators of circadian entrainment in the peripheral tissues (Pevet and Challet, 2011; Pezük et al., 2012). The circadian regulation of GC release is a result of close interaction between the SCN and clocks along the hypothalamus-pituitary–adrenal (HPA) axis (Oster et al., 2006; Son et al., 2008). Efferences from the SCN influence the release of corticotropin-releasing hormone (CRH) by the paraventricular nucleus of the hypothalamus (PVN), both inhibitory and stimulatory inputs from the SCN, depending on fluctuating arginine vasopressin (AVP) levels, are responsible of generating the complete daily profile of GCs (Kalsbeek et al., 1992, 1996; Buijs et al., 1993). The PVN controls the rhythmic secretion of adrenocorticotropic hormone (ACTH) from the pituitary and, consequently, GC production by the adrenal gland. Via autonomic pathways the SCN-PVN synchronize adrenal clocks regulating the time-of-day-dependent sensitivity of the adrenal to ACTH stimulation (Oster et al., 2006; Son et al., 2008). As a result, GCs are peaking at the beginning of the active phase to coordinate complex functions when high energy demands are expected (Balsalobre et al., 2000). For instance, GCs promote glucose and lipid mobilization through gluconeogenesis in the liver and the release of free fatty acids from adipocytes. GC rhythms are key regulators of mood and cognition, demonstrated by the improvement of learning skills during GC peak (Liston et al., 2013) and the impairment of memory retrieval when circadian GC rise is blocked pharmacologically (Rimmele et al., 2010).

Compared to the knowledge we have on the adult circadian system, little is known about its assembly and function during development. The view of the fetal hypothalamic clock as a “dependent” oscillator entrained by maternal rhythmic signals has been questioned by our own findings and by others (Landgraf et al., 2015; Astiz et al., 2020; Lužná et al., 2020; Greiner et al., 2022). In mice, the SCN circuit develops, matures, and connects to afferent and efferent pathways around birth (Munekawa et al., 2000; Sekaran et al., 2005; Kabrita and Davis, 2008). However, some early rhythmic genes of the molecular clock machinery, such as *RevErbα*, are already expressed in the hypothalamus earlier (Čečmanová et al., 2019; Astiz et al., 2020; Carmona-Alcocer et al., 2020). Since both, astrocytes and neurons, display strong circadian rhythmicity, it seems possible that the two could promote the gain of autonomous function of the developing SCN and participate in organizing the coupling between the SCN and the HPA axis, essential for the adaptation to the environment after birth (Astiz and Oster, 2018). Extensive evidence suggests that during this critical developmental window, the fetal/neonatal SCN and HPA axis are susceptible to GCs. An excess, a deficit or even the exposure at the wrong time of day can have persisting effects later on (Moisiadis and Matthews, 2014a; Busada and Cidlowski, 2017; Astiz et al., 2020).

## 2. Molecular interaction between Glucocorticoid receptors and the clock machinery

The strong coupling between the clock and the stress system is nourished by a solid bidirectional interaction at the molecular level. GCs activate, in target cells, intracellular GC receptors (GR) and mineralocorticoid receptors (MR). The primary ligands of GR are cortisol and other GCs (i.e., corticosterone, the main GC in rodents), while the primary ligand of MR is aldosterone in the periphery and corticosterone in the central nervous system (CNS) (Fuller et al., 2012). While GR is widely distributed within the body and especially abundant in the brain and the pituitary, MR is prominently present in peripheral tissues and in the brain, the expression is high in the magnocellular neurons and pre-sympathetic neurons of the hypothalamic paraventricular nuclei (PVN) as well as in the hippocampus and cerebral cortex (Oyamada et al., 2008; Fuller et al., 2019). Since MR has a 10-fold higher affinity for GCs than GR, it becomes active even at nadir GC levels (Kolber et al., 2008; Daskalakis et al., 2022). In contrast, GR is only activated at higher GC concentrations, such as those reached during the circadian peak or after stress-induced HPA axis activation (Kolber et al., 2008). The presence of both GR and MR in the neurons of the SCN of adult mice and humans remains a controversial (Rosenfeld et al., 1988; Olejníková et al., 2018). The genes encoding for GR and MR are not expressed in adult SCN neurons in rodents and humans.^1^

GR acts as a ligand-activated transcription factor. In the inactive/unbound state, GR locates in the cytosol bound to the N-terminal and middle region of a chaperone complex containing Heat shock protein 90 (HSP90) (Antunica-Noguerol et al., 2016; Fries et al., 2017; Mazaira and Galigniana, 2019). HSP90 does not form part of the active receptor complex, but is important for correct GR protein folding and stability (Biebl and Buchner, 2019). The HSP90 complex is also formed by co-chaperones important for GR stabilization and feedback, such as FK506-binding protein 5 (FKBP5) (Binder, 2009; Biebl and Buchner, 2019). *Fkbp5* is transcriptionally induced by GC-GR and, in turn, regulates GR sensitivity through an intracellular negative feedback loop, essentially keeping inactive GR in the cytosol (Pratt and Toft, 1997; Nicolaides et al., 2000; Binder, 2009; Klengel et al., 2013; Criado-Marrero et al., 2018). Upon ligand binding, GR monomers dimerize (either with other GR monomer or with MR) and translocate to the nucleus where the dimer binds the DNA at GC response elements (GREs), regulatory regions of target genes (George et al., 2017; Oakley et al., 2021). Through binding of GREs, the transcription of GR target genes is modulated positively or negatively (Reddy et al., 2009; Surjit et al., 2011). Besides this canonical signaling cascade, GR and MR activation have shown rapid non-genomic changes, mediated by membrane-anchored GR/MR that might mediate the coordination of rapid adaptive responses to stress (Groeneweg et al., 2012).

**Figure 1:**
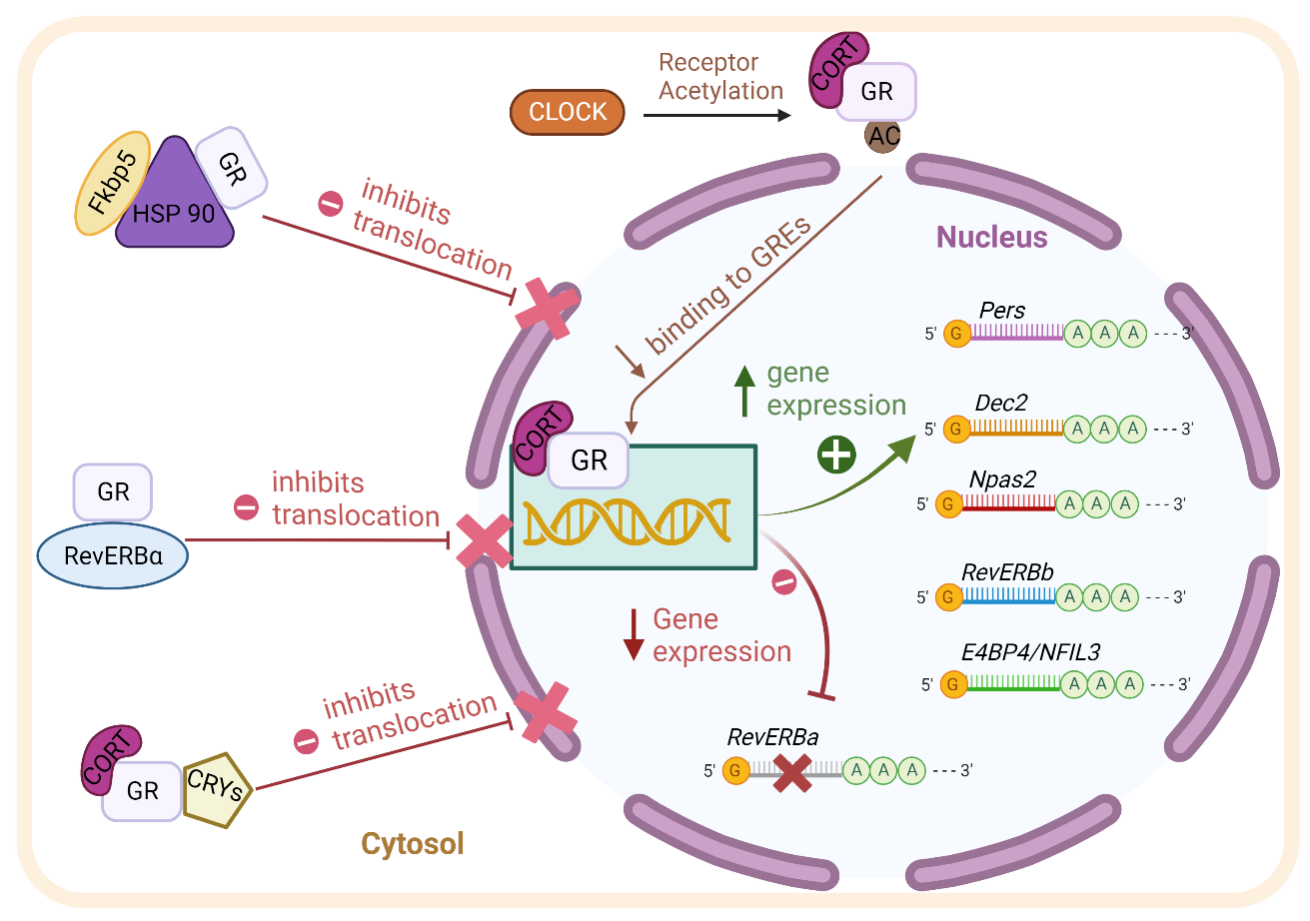
Scheme of the reciprocal molecular interaction between GR and the clock machinery at transcriptional, translational, and post-translational levels as described in detail in the text. BMAL1: brain and muscle aryl hydrocarbon receptor nuclear translocator-like 1. CLOCK: circadian locomotor output cycles kaput. *Pers: Periods. Dec2: differentially expressed in Chondrocytes 2. Npas2: Neuronal PAS Domain-Containing Protein 2. RevErb-beta: Nuclear Receptor Subfamily 1 Group D Member 2.* AC: acetyl group. GR: Glucocorticoid receptor. RevERb-Alpha: Nuclear Receptor Subfamily 1 Group D Member 1. CRY1/2 (Cryptochrome 1/2). HSP90: Heat shock protein 90. FKBP5: FK506-binding protein 5. CORT: Corticosterone.

The clock gene machinery is present in almost every cell of the body and reciprocal interaction with GR is found at transcriptional, translational, and post-translational levels (summarized in Figure 1; Nader et al., 2010). The molecular clock machinery works as an interlocked transcriptional-translational-feedback loop (Takahashi, 2017). The positive members of the loop: BMAL1 (brain and muscle aryl hydrocarbon receptor nuclear translocator-like 1) and CLOCK (circadian locomotor output cycles kaput) heterodimerize and activate the transcription of their repressors *Per1/2* (*Period 1/2*), *Cry1/2* (*Cryptochrome 1/2*) through binding of E-Box promoter elements. Upon translation, PER and CRY form complexes in the cytosol, translocate into the nucleus and repress CLOCK/BMAL1 activity, shutting down their own transcription. After clearance of PER/CRY complexes, the inhibition of CLOCK/BMAL1 is released and a new circadian (~24 h) cycle begins. The cycle is further stabilized by accessory loops in which CLOCK/BMAL1 drive the circadian expression of Dec 1/2 and nuclear receptors of the ROR and the REV-ERB family (Takahashi, 2017).

As mentioned above, GR through its ability to respond to GCs’ circadian oscillations, is an ideal coupling partner of the molecular clock (Lambrou et al., 2021). Activated GR is able to influence the transcription of various clock genes via binding to GREs in their promoter regions, among them *Per2, Rev-ERBß, Dec2, NPAS2,* and *E4BP4/NFIL3* (So et al., 2009; Nader et al., 2010). The clock gene *Per1* contains both, E-Box and GRE elements in its promoter; it is therefore responsive to both, BMAL1/CLOCK and GR binding (Conway-Campbell et al., 2010). Moreover, the nuclear receptor *Rev-ERBα* is repressed by GR activity through negative GREs present in its promoter (Surjit et al., 2011).

The clock machinery and GR also interact at the protein level. It has been shown that Rev-ERBα, GRs and HSP90 interact in the cytosol (Okabe et al., 2016). Moreover, Rev-ERBα and GR distribute differentially between the nuclei and the cytosol depending on the phase of the clock, suggesting a possible influence of each other with regard to intracellular trafficking (Surjit et al., 2011; Okabe et al., 2016). The above-mentioned interaction has been shown in cultured fibroblast, whether this mechanism is also present in brain cells needs to be determined. CLOCK harbors a histone-acetyl-transferase activity which is able to alter the affinity of GR to GREs through acetylation of lysine clusters in its hinge region (Nader et al., 2010). Also, CRY proteins are able to decrease GR’s transactivation potential by interacting directly with GR (Lamia et al., 2011). These multiple and reciprocal molecular interactions stress the relevance of both pathways for the cellular and systemic regulation of circadian rhythms.

## 3. Glucocorticoids during circadian system development

During pregnancy, maternal physiology including the circadian system has to adapt to fulfill fetal requirements. During the first two thirds of pregnancy, the embryo/fetus is protected by the placenta from excessive levels of GCs by expressing (among others) the enzyme 11β-Hydroxysteroid dehydrogenase (11β-HSD2) which inactivates GCs converting them into an inactive metabolite, cortisone (O’Donnell et al., 2012). Even though only 10% of maternal GCs reach the fetus, a circadian rhythmicity of GCs can still be detected in fetal blood (Astiz et al., 2020). During the last third of pregnancy, there is a gradual increase of GC levels in fetal blood (Atkinson and Waddell, 1995) that helps the fetus to continue developing and growing (Fowden et al., 2022). This is partly due to a down-regulation of 11β-HSD2 (Wieczorek et al., 2019). Therefore, even if GC concentration changes over pregnancy, the circadian rhythmicity is always present, and since GR is expressed at high levels in the fetal hypothalamus (including the SCN during that period), it could be a key signal driving the development and maturation of the circadian system and its coupling to the HPA axis (Astiz et al., 2020; Greiner et al., 2022).

Indeed, extensive evidence shows that maternal stress or circadian rhythm disruption (e.g., through altered photoperiod, sleep deprivation, shift work) leads to a higher risk of developing metabolic, behavioral, and sleep disorders later in life (Moisiadis and Matthews, 2014b; Vilches et al., 2014; Marín, 2016; Mendez et al., 2016; Logan and McClung, 2019; Lužná et al., 2020). Interestingly, due to the close SCN-HPA axis relationship most of the experimental paradigms assessing the long-term effects of maternal stress used in nocturnal rodents entail some degree of circadian disruption because animals are subjected to different manipulations during their normal rest/light phase. Reciprocally, circadian disruption paradigms entail a certain degree of activation of the stress system. This issue was disentangled recently by our lab, when we demonstrated that the offspring from mothers exposed to GCs during the rest phase show worse circadian and stress-related behavioral phenotypes than those from mothers exposed to the same GC concentration, but during the active phase. This means that the effect of prenatal GCs activating epigenetic mechanisms and programming offspring behavior depends on the maternal exposure time (Astiz et al., 2020). We have found that when GC exposure happens, GR in the fetal hypothalamus is activated only if the signal reaches the tissue during the inactive phase. This time-dependent sensitivity to GCs in the fetal hypothalamus was gated by an autonomous function of the fetal molecular clock (Astiz et al., 2020). Then, the maturation of the clock and stress axis of the newborn seems to depend not only on maternal GCs but also on GR sensitivity, which is time-dependent.

## 4. Glucocorticoids and the adult circadian system

From a physiological point of view, an efficient synchronization of the circadian system would require that central or peripheral signals feedback to the SCN to adjust its function according to the internal environment. In the adult, SCN hypothalamic efferences reach the sub-PVN, PVN, supraoptic nuclei (SON), dorsomedial nucleus (DMH), and the arcuate nucleus (ARC) among many others (Saeb-Parsy et al., 2000; Kalsbeek et al., 2004; Postnova et al., 2013; Buijs et al., 2017) and some of those connections are reciprocal (e.g., with the ARC). However, the SCN clock is quite resistant to re-setting signals. This property, so far attributed to the robust network feature of the SCN circuit, might be important to prevent the impact of *Zeitgeber* noise such as sporadic light exposure, acute stress, or miss-timed food intake on the central pacemaker (Schibler et al., 2015).

GCs, as one of the main outputs of the circadian system, might feedback to the SCN, however, it is still under discussion whether the influence is direct or indirect (Balsalobre et al., 2000). In the early 90s, it was demonstrated that SCN neurons are among the few cell types in adult rodents that do not express GR (Rosenfeld et al., 1988). Interestingly, experiments manipulating GC circadian phase have shown that, GCs have a key role in circadian resynchronization of locomotor activity after jetlag, indicating a clear influence on the SCN (Kiessling et al., 2010). Furthermore, either the removal of the adrenal glands or the restitution of the hormone within physiological limits in adrenalectomized rats, demonstrated that glucocorticoids are involved in plastic rearrangements of the SCN circuit (Maurel et al., 2000). These ultrastructural arrangements have been observed over the 24 h cycle characterized by day/night changes of the glial, axon terminal, and/or somato-dendritic coverage of neurons expressing arginine vasopressin (AVP) or vasoactive intestinal peptide (VIP) (Becquet et al., 2008).

**Figure 2:**
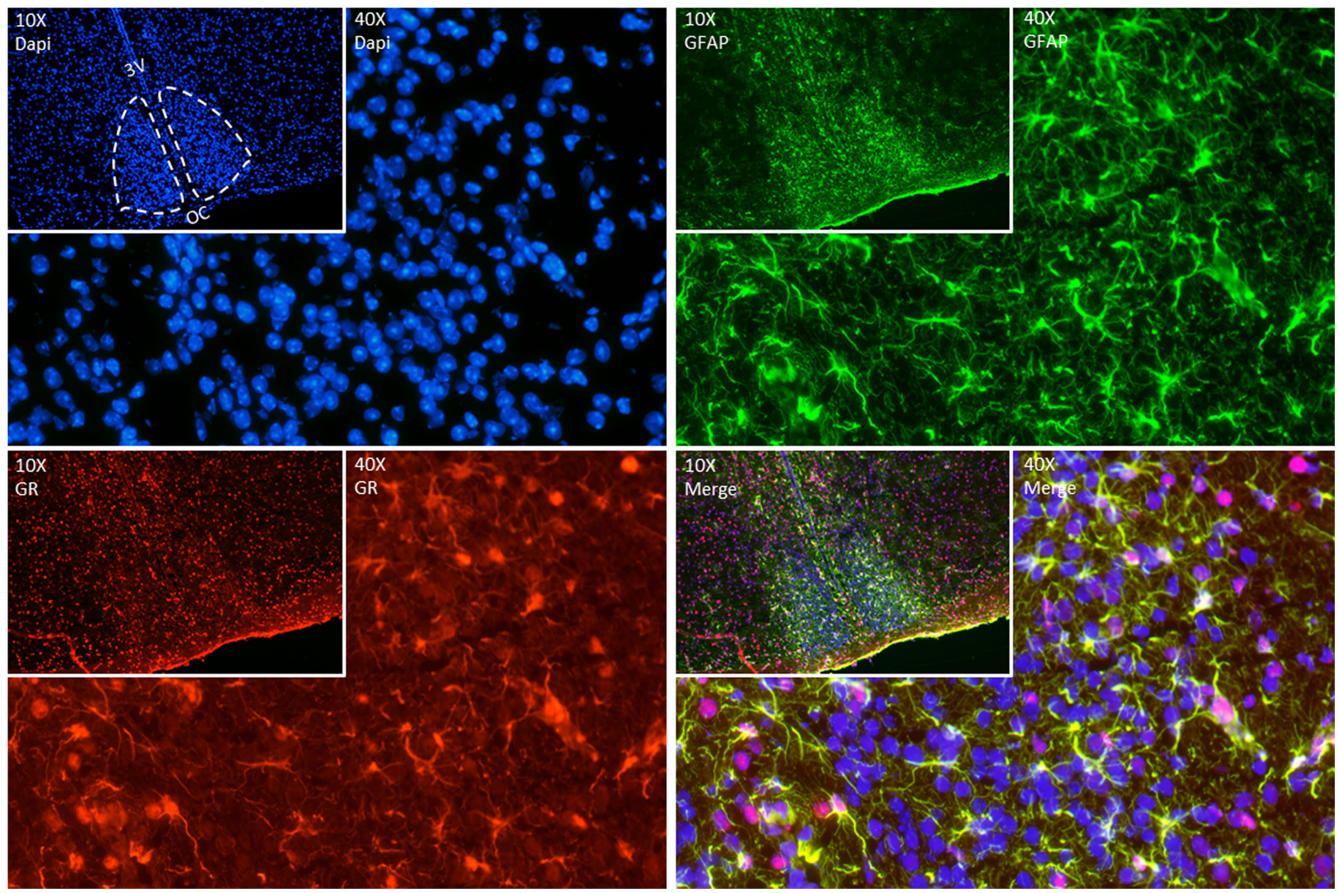
Representative double fluorescence immunostainings in the adult SCN. GFAP (green) used as an astrocyte marker, GR (red) and DAPI (blue) as a nuclear marker. Brain was collected from a 90-day old mouse at ZT (Zeitgeber time) 7, immediately frozen on dry ice, and sectioned (12 μm) in a cryostat. After fixation (20 min at room temperature (RT)), sections were blocked with goat serum 4% in phosphate buffer (PBS) containing 0.4% of Triton X-100 for 1 h at RT. Sections were incubated with anti-GR (Abcam Ab183127, 1:200 in blocking) for 2 nights at 4°C. Sections were incubated afterwards with anti-GFAP (ThermoFisher 14–9,892-82, 1:200 in blocking) for 1 night at 4°C. The day after, sections were incubated with secondary antibodies – goat anti-rabbit Alexa 594 (ThermoFisher A11012, 1:500) and goat anti-mouse Alexa 488 (ThermoFisher A11029, 1:500) in a dark chamber for 2 h at RT. DAPI staining was performed to stain nuclei by incubating slides with 300 nM DAPI (ThermoFisher D1306) in PBS for 2 min in the dark. Images of immunofluorescent staining were made using Nikon Ts2R-FL (10X (insets) and 40X magnification).

As mentioned before, we know now that astrocytes play a key role as time-keepers of the adult SCN. Astrocytes express the molecular clock machinery with self-sustained circadian rhythms that persist even in constant environmental conditions (Prolo et al., 2005; Marpegan et al., 2009; Barca-Mayo et al., 2017). The molecular clock in SCN astrocytes is entrainable by neuronal signals (Marpegan et al., 2009), temperature changes (Prolo et al., 2005), and they reciprocally modulate neuronal rhythms (Barca-Mayo et al., 2017). Additionally, several astroglial functions are under circadian control such as, intracellular Ca^2+^ waves (Brancaccio et al., 2017) and the release of gliotransmitters, ATP (Burkeen et al., 2011) and glutamate (Svobodova et al., 2018) and even their morphological changes during the day (Becquet et al., 2008). Interestingly, we have found GR expression in the SCN of adult mice colocalizing with glial fibrillary acidic protein (GFAP) a marker of mature astrocytes (Figure 2) confirming previous observations (Moriya et al., 2000; Leone et al., 2006). Although this needs further investigation it opens the possibility that not only neurons but also astrocytes might play an essential role in the complex cooperation between the SCN, the HPA axis and the systemic coordination of circadian rhythms.

## 5. Perspective

In summary, accumulating evidence suggests that GCs play an important role in the development and maturation of the circadian system. This, in turn, feeds back on stress axis regulation in the adult. Glial clocks may play an important role in this context, but experimental evidence for this is still sparse. To causally address such interaction, it would be important to study clock system development in animals with alterations in glial clock function and how glial disruption affects clock and stress regulation in the offspring. With regard to humans, the relevance of perinatal rhythms for shaping circadian system regulation and stress responses offers tremendous potential for therapeutic interventions. Can stabilization of GC rhythms during the end of pregnancy lead to increased resilience of the offspring against stress and stress-associated disorders, and what type of interventions beyond GC treatment are efficient in this context? To which extent is GC perinatal programming reversible later in life, and—against the background of the broad impact of stress hormones on physiological functions—what other systems are affected? Finally, how does GC programming affect glial function later in life, and what does this mean for neuro-immunologic disorders? First steps in this direction have been taken with the studies on perinatal GC treatments (Astiz et al., 2020). It will be interesting to further decipher the underlying mechanisms of GC-fetal rhythm interaction and search for additional factors that impinge on this crosstalk.

## Author contributions

All authors listed have made a substantial, direct, and intellectual contribution to the work and approved it for publication.

## Funding

This work was supported by the German Research Foundation (DFG) grant AS547-1/2 (to ML and MA) and OS353-10/1 (to HO), by Spanish Research Grants PID2021-122694OB-I00 funded by Ministerio de Ciencia e Innovacion (MCIN) to MA and KH, by Achucarro Basque Center for Neuroscience and Basque Foundation of Science (Ikerbasque) to MA, and by the Federation of the European Biochemical Societies (FEBS) Summer Fellowship 2022 (to KH).

## Conflict of interest

The authors declare that the research was conducted in the absence of any commercial or financial relationships that could be construed as a potential conflict of interest.

## Publisher’s note

All claims expressed in this article are solely those of the authors and do not necessarily represent those of their affiliated organizations, or those of the publisher, the editors and the reviewers. Any product that may be evaluated in this article, or claim that may be made by its manufacturer, is not guaranteed or endorsed by the publisher.

## Footnotes

^1^https://portal.brain-map.org/
